# Metabolomic Profiling of Pompe Disease‐Induced Pluripotent Stem Cell‐Derived Cardiomyocytes Reveals That Oxidative Stress Is Associated with Cardiac and Skeletal Muscle Pathology

**DOI:** 10.5966/sctm.2015-0409

**Published:** 2016-08-18

**Authors:** Yohei Sato, Hiroshi Kobayashi, Takashi Higuchi, Yohta Shimada, Hiroyuki Ida, Toya Ohashi

**Affiliations:** ^1^ Department of Pediatrics, Jikei University School of Medicine, Tokyo, Japan; ^2^ Division of Gene Therapy, Research Center for Medical Sciences, Jikei University School of Medicine, Tokyo, Japan

**Keywords:** Pompe disease, Pluripotent stem cell, Metabolomic profiling, Oxidative stress, Mitochondrial dysfunction

## Abstract

Pompe disease (PD) is a lysosomal storage disease that is caused by a deficiency of the acid α‐glucosidase, which results in glycogen accumulation in the lysosome. The major clinical symptoms of PD include skeletal muscle weakness, respiratory failure, and cardiac hypertrophy. Based on its severity and symptom onset, PD is classified into infantile and late‐onset forms. Lysosomal accumulation of glycogen can promote many types of cellular dysfunction, such as autophagic dysfunction, endoplasmic reticulum stress, and abnormal calcium signaling within skeletal muscle. However, the disease mechanism underlying PD cardiomyopathy is not fully understood. Several researchers have shown that PD induced pluripotent stem cell (iPSC)‐derived cardiomyocytes successfully replicate the disease phenotype and are useful disease models. We have analyzed the metabolomic profile of late‐onset PD iPSC‐derived cardiomyocytes and found that oxidative stress and mitochondrial dysfunction are likely associated with cardiac complications. Furthermore, we have validated that these disease‐specific changes were also observed in the cardiomyocytes and skeletal muscle of a genetically engineered murine PD model. Oxidative stress may contribute to skeletal muscle and cardiomyocyte dysfunction in PD mice; however, NF‐E2‐related factor 2 was downregulated in cardiomyocytes and skeletal muscle, despite evidence of oxidative stress. We hypothesized that oxidative stress and an impaired antioxidative stress response mechanism may underlie the molecular pathology of late‐onset PD. Stem Cells Translational Medicine *2017;6:31–39*

**Significance Statement**

Pompe disease (PD) is a lysosomal storage disease that is caused by a deficiency of the acid α‐glucosidase, which results in glycogen accumulation in the lysosome. An analysis of the metabolomic profile of late‐onset PD induced pluripotent stem cell‐derived cardiomyocytes found that oxidative stress and mitochondrial dysfunction are likely associated with cardiac complications. Furthermore, these disease‐specific changes were also observed in the cardiomyocytes and skeletal muscle of a genetically engineered murine PD model. Oxidative stress may contribute to skeletal muscle and cardiomyocyte dysfunction in PD mice; however, NF‐E2‐related factor 2 was downregulated in cardiomyocytes and skeletal muscle, despite evidence of oxidative stress.

## Introduction

Pompe disease (PD) is an autosomal recessive lysosomal disorder that is caused by a deficiency of the acid α‐glucosidase (GAA) 1. Systemic accumulation of glycogen induces progressive muscular weakness, respiratory failure, and hypertrophic cardiomyopathy. We have previously reported on PD disease modeling and gene transfer of late‐onset PD‐induced pluripotent stem cell (iPSC)‐derived cardiomyocytes 2. The cellular pathology of PD is still unknown; however, some mechanisms, such as autophagic buildup and endoplasmic reticulum stress, have been shown to be associated with disease progression and the development of refractive disease against enzyme‐replacement therapy, particularly in skeletal muscle 3, 4.

PD is a monogenic disease that is caused by GAA deficiency and dysregulation of glycogen metabolism 5. We hypothesized that glycogen accumulation may affect the cellular metabolism, including glycolysis and oxidative phosphorylation (OXPHOS). Huang et al. previously reported that PD iPSCs are characterized by abnormal energy metabolism using the Extracellular Flux Analyzer and showed that cellular energy metabolic processes, such as glycolysis and OXPHOS, are decreased in PD iPSCs compared with wild‐type (WT) iPSCs 6. To investigate cellular metabolism in a PD model, we acquired cardiomyocytes differentiated from late‐onset PD iPSCs and analyzed their metabolomic profile by liquid chromatography‐mass spectrometry (LC‐MS) and capillary electrophoresis‐mass spectrometry (CE‐MS).

Moreover, we validated the disease‐specific dysregulation of metabolomic processes, including the increased oxidative stress that was observed in the skeletal muscle and cardiomyocytes of PD model mice. We have also evaluated antioxidative stress mechanisms, such as NF‐E2‐related factor 2 (NRF‐2), in the skeletal muscle and cardiomyocytes of PD model mice.

## Materials and Methods

### Pompe Disease iPSCs and Cardiomyocytes

PD iPSCs (HPS0175) were kindly provided by RIKEN BioResource Center (BRC) 7. Control iPSCs (HPS0223) were also kindly provided by RIKEN BRC 8. The iPSCs were analyzed by immunofluorescence (IF) of pluripotency markers. Immature markers, such as SSEA4, Tra‐1‐60, and Tra‐1‐81, were analyzed by an embryonic stem cell characterization kit (Miltenyi Biotec, San Diego, CA, http://www.miltenyibiotec.com). The levels of transcription factors, such as Oct4, Sox‐2, and Nanog, were analyzed by using the StemLight Pluripotency Transcription Factor Antibody Kit (Cell Signaling Technology, Beverly, MA, http://www.cellsignal.com). G‐band karyotype analyses of the PD and control iPSCs were conducted to evaluate chromosome status (Nihon Gene Research Laboratories, Sendai, Japan, http://www.ngrl-japan.com).

The differentiation of the PD and control iPSCs into cardiomyocytes was performed as described previously 9. Cardiac troponin T was stained by using a fluorescein isothiocyanate (FITC)‐conjugated anti‐cardiac troponin T (anti‐cTnT) antibody (Miltenyi Biotec) and analyzed by fluorescence‐activated cell sorting (FACS) to qualify cardiac differentiation (*n* = 3). IF of the iPSC‐derived cardiomyocytes was conducted by using the anti‐cTnT antibody (mouse monoclonal antibody; Thermo Fisher Scientific Life Sciences, Oakwood Village, OH, https://www.thermofisher.com; 1:100) and anti‐GAA antibody (rabbit polyclonal antibody; Sigma‐Aldrich, St. Louis, MO, http://www.sigmaaldrich.com; 1:100). An Alexa Flour 488 anti‐mouse secondary antibody and Alexa Flour 568 anti‐rabbit secondary antibody were also used (Thermo Fisher 1:1,000). Nuclei were stained with 4′,6‐diamidino‐2‐phenylindole (Dojindo Molecular Technologies, Rockville, MD, http://www.dojindo.com).

### CE‐MS Analysis

Metabolites were extracted from both the PD‐ and the control‐differentiated cardiomyocytes (*n* = 3). In total, 2,000,000–2,500,000 cells were washed with 5% mannitol that was dissolved in ultrapure water, and the metabolites were extracted with methanol before being purified by ultrafiltration. The cations and anions were measured by CE‐MS. All detected peaks were annotated according to a metabolite library (Human Metabolome Technologies, Boston, MA, http://humanmetabolome.com).

### LC‐MS Analysis

Metabolites were extracted from both the PD‐ and the control‐differentiated cardiomyocytes (*n* = 3). In total, 2,250,000–2,500,000 cells were washed with 5% mannitol that was dissolved in ultrapure water. These cells were detached by scraping and collected in ethanol. The metabolites were extracted by sonication, and the supernatant containing the metabolites was collected after centrifugation. The metabolites were measured by both positive‐ and negative‐ion mode LC‐MS. All detected peaks were annotated according to a metabolite library (Human Metabolome Technologies).

### Statistical Analysis

For the metabolome analyses, PCA was conducted by using SampleStat (version 3.14; Human Metabolome Technologies), and hierarchical clustering analysis was conducted by PeakStat (version 3.18, Human Metabolome Technologies). Each metabolite was compared by Welch's *t* test. Other statistical calculations comparing the results between two groups for FACS, glutathione, and Western blot analyses were conducted by Student's *t* test using GraphPad Prism (version 5.0; GraphPad Software, La Jolla, CA, http://www.graphpad.com).

### Animals

The PD model (B6; 129‐Gaa^tm1Rabn^/J) was kindly provided by Dr. Raben at the NIH. C57BL/6J mice were used as a wild‐type control. The PD and WT mice were sacrificed between 16 and 20 weeks, and cardiomyocytes and skeletal muscle were harvested for further analysis. All animal experiments were approved by The Jikei University School of Medicine's animal experimental committee (2015‐050).

### Western Blot

Proteins were extracted from mouse tissue by homogenization into SDS sample buffer containing a protease inhibitor cocktail (Roche Life Science, Indianapolis, IN, https://lifescience.roche.com). The proteins were isolated by SDS‐polyacrylamide gel electrophoresis and transferred to a nitrocellulose membrane. The primary antibodies that were used included anti‐NRF‐2 (mouse monoclonal; MBL, Woburn, MA, https://www.mblintl.com), anti‐KEAP‐1 (rabbit polyclonal; Cell Signaling Technology), anti‐GAA (mouse monoclonal; Sanofi Genzyme, Cambridge, MA, https://www.genzyme.com), anti‐AMP‐activated protein kinase (anti‐AMPK)/*p*‐AMPK α and β subunits (rabbit monoclonal; Cell Signaling Technology), and anti‐actin (mouse monoclonal; Sigma‐Aldrich) antibodies. The primary antibodies (1:1,000) were incubated overnight, and the secondary antibodies (anti‐mouse and rabbit; Nichirei Bioscience, Tokyo, Japan, https://www.nichirei.co.jp; 1:10,000) were incubated for 2 hours. The proteins were visualized with ImmunoStar LD (Wako Pure Chemical Industries, Ltd., Osaka, Japan, http://www.wako-chem.co.jp), and fluorescence was analyzed by ChemiDoc XRS (Bio‐Rad, Hercules, CA, http://www.bio-rad.com).

### Glutathione Redox Ratio Assay

Total glutathione (GSH + GSSG) and reduced glutathione (GSH) levels were measured by the Total Glutathione Assay Kit (Cell Biolabs, San Diego, CA, http://www.cellbiolabs.com). Briefly, the cell and tissue lysates were dissolved in assay buffer, and the total amount of GSH was measured with or without glutathione reductase, which converts GSSG into GSH (*n* = 3). Oltipraz (Sigma‐Aldrich), which is a NRF‐2 activator working as an antioxidant, was added to the medium 24 hours before the measurement at concentrations of 10 or 50 μM 10.

### Reactive Oxygen Species Assay

Cardiomyocytes derived from iPSCs were stained with CellRox (Thermo Fisher) for reactive oxygen species (ROS) visualization, according to the manufacturer's instructions. Oltipraz (Sigma‐Aldrich) was also added to the medium 24 hours before the measurement at concentrations of 10 or 50 μM.

## Results

### Characterization of Pompe Disease iPSCs and Cardiomyocytes

**Figure 1:**
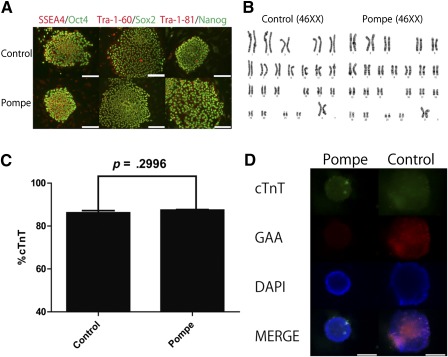
Characterization of Pompe disease induced pluripotent stem cells (iPSCs) and cardiac differentiation. **(A):** Immunofluorescence of iPSCs (control and Pompe) was used to analyze SSEA4/Oct4, Tra‐1‐60/Sox2, and Tra‐1‐81/Nanog expression. Scale bars = 100 µm**. (B):** Karyotyping analysis was used to show normal human karyotypes (46XX). **(C):** cTnT expression was measured by fluorescence‐activated cell sorting (mean + SEM; *n* = 3). **(D):** Immunofluorescence of iPSC‐derived cardiomyocytes for cTnT (Alexa 488) and GAA (Alexa 568) was analyzed. Nuclei were stained with DAPI. Scale bars = 100 µm. Abbreviations: cTnT, cardiac troponin T; DAPI, 4′,6‐diamidino‐2‐phenylindole; GAA, acid α‐glucosidase.

The expressions of pluripotency markers, such as SSEA‐4, Tra‐1‐60, and Tra‐1‐81, and transcription factors, such as SOX‐2, OCT4, and Nanog, were confirmed by immunofluorescence (Fig. 1A). Karyotype analysis showed that both the control and the PD iPSCs harbored a normal female human karyotype (Fig. 1B). Both the PD and the control iPSCs displayed similar expression levels of the pluripotent markers indicated above.

We generated cardiomyocytes using a robust differentiation protocol that was previously described using a Wnt inhibitor 9. Differentiated cardiomyocytes were validated by cTnT expression, as measured by FACS analysis (Fig. 1C). PD iPSC‐derived cardiomyocytes have similar characteristics to control iPSC‐derived cardiomyocytes, including cTnT expression, but not GAA expression, as shown by IF (Fig. 1D). After cardiac differentiation, the cardiomyocytes were collected, and the metabolites were extracted for LC‐MS and CE‐MS analysis.

### Metabolomic Profiling of iPSC‐Derived Cardiomyocytes by CE‐MS

**Figure 2:**
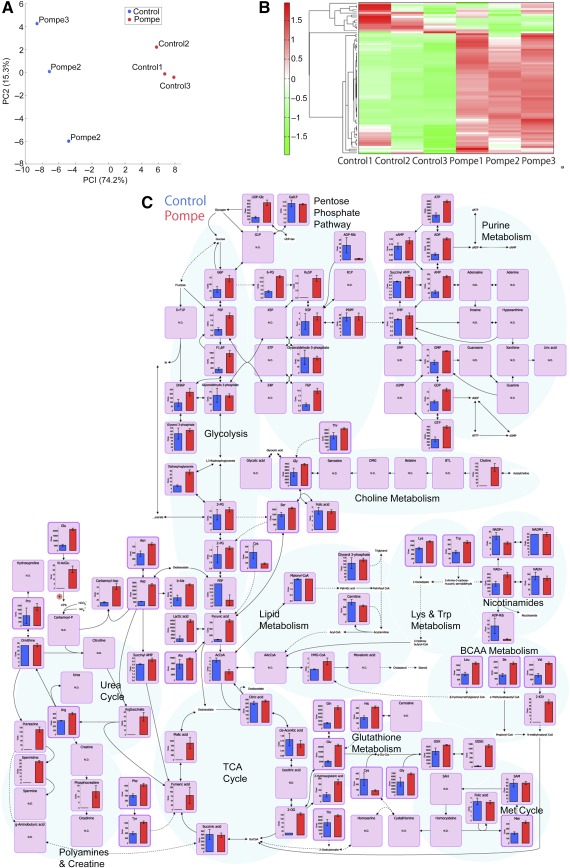
Capillary electrophoresis‐mass spectrometry analysis of induced pluripotent stem cell‐derived cardiomyocytes. **(A):** Principal component analysis of metabolites. **(B):** Clustering and heatmap analysis of metabolites. **(C):** A whole map of metabolites (mean + SD; *n* = 3.) Abbreviations: BCAA, branched‐chain amino acid; PC2, principal component 2; TCA, tricarboxylic acid.

A total of 116 metabolites (52 cations and 64 anions) were detected by CE‐MS ( supplemental online Table 1). Principal component analysis (PCA) revealed that the metabolites from PD iPSC cardiomyocytes display unique features (Fig. 2A). Cluster analysis was also conducted to compare the metabolite profiles of cardiomyocytes derived from PD or control iPSCs (Fig. 2B). An entire map of metabolites is shown in Figure 2C. The PD iPSC‐derived cardiomyocytes displayed a unique metabolite pattern characterized by higher amino acid and lactate levels and GSSG content, as well as lower carnitine content, compared with the control iPSC cells. These results suggest that some metabolic pathways may be involved in the pathology of PD.

### Pompe Disease iPSC‐Derived Cardiomyocytes Display Differential Oxidative Stress‐Associated Metabolic Parameters

**Figure 3:**
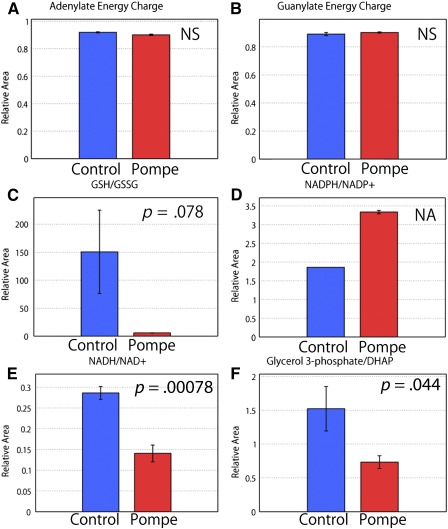
The metabolic parameters of induced pluripotent stem cell‐derived cardiomyocytes calculated by capillary electrophoresis‐mass spectrometry analysis. Levels of adenylate energy charge **(A)**, guanylate energy charge **(B)**, GSH/GSSG **(C)**, NADPH/NADP+ **(D)**, NADH/NAD+ **(E)**, and glycerol 3‐phosphage/DHAP **(F)** were assessed as described in Materials and Methods (mean + SD; *n* = 3). Abbreviations: DHAP, dihydroxyacetone phosphate; GSH, reduced glutathione; GSSG, oxidized glutathione; NA, not applicable; NS, not significant.

To evaluate the cellular metabolomic status of differentiated cardiomyocytes, we calculated their metabolic parameters ( supplemental online Table 2). The adenylate and guanylate energy charges were calculated, and no significant differences were observed between the PD and the control iPSC‐derived cardiomyocytes (Fig. 3A, 3B), suggesting that the energy statuses of the two cell types were similar and that energy disruption was not observed in either type of cardiomyocyte.

Then, we calculated the glutathione redox ratio, which is indicative of oxidative stress in the cell (Fig. 3C). The PD iPSC‐derived cardiomyocytes showed a remarkable decrease in their GSH/GSSG ratio, which was suggestive of oxidative stress. In addition, the NADPH/NADP^+^ ratio, which plays a role in preventing cellular oxidative damage, was elevated in the PD iPSC‐derived cardiomyocytes (Fig. 3D). The NADPH/NADP^+^ ratio for glucose‐6‐phospate dehydrogenase (G6PD) activity was also analyzed, and elevated levels of G6PD were suspected, which is in line with previous results indicating that G6P levels are increased in the cardiomyocytes of PD model mice. Dysregulation of glycogen metabolism has been suspected in PD iPSC‐derived cardiomyocytes.

The NADH/NAD^+^ and glycerol 3‐phosphate/dihydroxyacetone phosphate ratios were calculated to determine whether energy production in oxidative phosphorylation was decreased in the PD iPSC derived cardiomyocytes (Fig. 3E, 3F). Huang et al. characterized infantile PD iPSCs with a reduced oxygen consumption rate and mitochondrial dysfunction 6. Our data are in line with those of previous reports.

### Metabolomic Profiling of iPSC‐Derived Cardiomyocytes by LC‐MS

**Figure 4:**
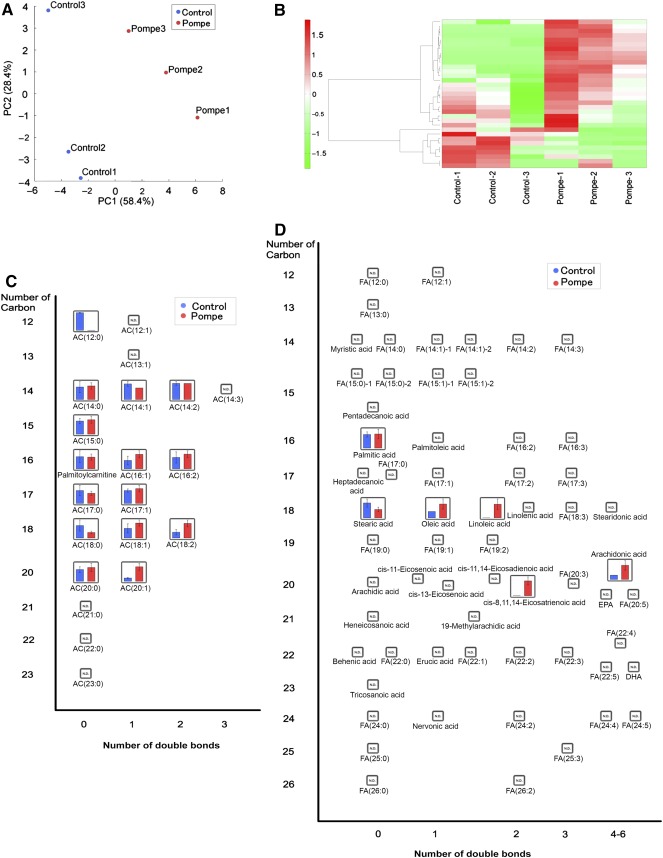
Liquid chromatography‐mass spectrometry analysis of induced pluripotent stem cell‐derived cardiomyocytes. **(A):** PCA of metabolites. **(B):** Clustering and heatmap analysis of metabolites. **(C):** A whole map of metabolites identified by our analysis (mean + SD; *n* = 3). **(D** **):** A whole map of fatty acid metabolites (mean + SD; *n* = 3). Abbreviations: AC, acylcarnitine; FA, fatty acid; PC2, principal component 2.

A total of 33 metabolites (27 positive and 6 negative) were detected by LC‐MS ( supplemental online Table 3). PCA showed that the metabolites from the PD iPSC cardiomyocytes displayed unique features (Fig. 4A). Cluster analysis was used to investigate the differences between the metabolites in the PD and control iPSC‐derived cardiomyocytes (Fig. 4B). Mapping of all detected metabolites (Fig. 4C) revealed differential profiles for acylcarnitine and fatty acids in the PD iPSC‐derived cardiomyocytes. Compared with the control iPSC‐derived cardiomyocytes, the PD iPSC‐derived cardiomyocytes had higher unsaturated long‐chain fatty acids (FA 18:1 and FA 18:2) and unsaturated long‐chain acylcarnitine (AC 18:1, AC 18:2, and AC 20:1) profiles, which was suggestive of β‐oxidation dysfunction. Decreased β‐oxidation could result from mitochondrial dysfunction and was also in line with the increased oxidative stress observed by CE‐MS analysis.

### PD Murine Cardiomyocytes and Skeletal Muscles Display Oxidative Stress

**Figure 5:**
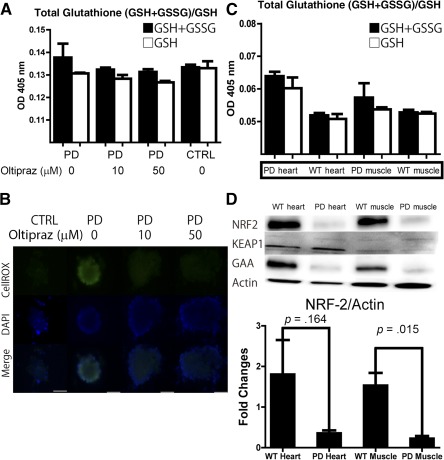
Oxidative stress analysis of PD induced pluripotent stem cell (iPSC)‐derived cardiomyocytes and PD model mice. **(A):** A glutathione assay was used to analyze iPSC‐derived cardiomyocytes. As indicated, Oltipraz was added at a final concentration of 10 or 50 µM (mean + SEM; *n* = 3). **(B):** A reactive oxygen species (ROS) assay was used to analyze iPSC‐derived cardiomyocytes. CellRox was added to the medium to visualize ROS production. Scale bars = 100 µm. **(C):** A glutathione assay was used to analyze the cardiomyocytes and skeletal muscle cells from PD mice (mean + SEM; *n* = 3). **(D):** Western blot analysis of the cardiomyocytes and skeletal muscle cells from PD mice (mean + SEM; *n* = 3). Abbreviations: CTRL, control; DAPI, 4′,6‐diamidino‐2‐phenylindole; GAA, acid α‐glucosidase; GSH, reduced glutathione; GSSG, oxidized glutathione; OD, optical density; PD, Pompe disease; WT, wild type.

CE‐MS metabolomic profiling suggests that oxidative stress may be linked to the cardiac complications associated with PD. Here, we validated the metabolomic changes in iPSC‐derived cardiomyocyte by oxidative stress measurements. First, total glutathione (GSSG+GSH) and GSH were measured by a glutathione assay (Fig. 5A). Then, we used CellRox Reagents to show that cardiomyocytes derived from PD iPSCs display evidence of oxidative stress (Fig. 5B). NRF‐2 signaling has been associated with oxidative stress and cell damage. We assessed the protein levels of NRF‐2 and actin ( supplemental online Fig. 1A) by using Western blot analysis. NRF‐2 was downregulated in the PD iPSC cardiomyocytes. This result confirms that the oxidative stress suggested by the metabolome analysis occurs in vitro.

Next, we investigated oxidative status in the cardiomyocytes and skeletal muscles of PD model mice. We have measured total glutathione (GSH+GSSG) and GSH in the cardiomyocytes and skeletal muscle of PD model mice (Fig. 5C). Compared with WT mice, the level of GSSG was assumed to increase in PD murine cardiomyocytes and skeletal muscle. Indeed, oxidative stress was shown to be more prominent in skeletal muscle cells than in cardiomyocytes.

To investigate NRF‐2 signaling in PD‐associated cardiomyocytes and skeletal muscle cells, we assessed the protein levels of NRF‐2, Keap‐1, GAA, and actin (Fig. 5D) using Western blot analysis. Compared with the WT mice, NRF‐2 was significantly downregulated in the PD murine cardiomyocytes and skeletal muscle cells, despite the increased oxidative stress observed in the GSH/GSSG assay (Fig. 5E). Together, these results suggest that the NRF‐2/ARE pathway is downregulated in the cardiomyocytes and skeletal muscle cells of PD model mice and that oxidative stress is not completely eradicated by the activation of antioxidative stress elements.

AMPK signaling maintains cellular energy metabolism and also involved in cellular stress. AMPK ( supplemental online Fig. 1A, 1B) and *p*‐AMPK (data not shown) were not changed in neither PD iPSC cardiomyocytes nor the PD murine cardiomyocyte in Western blot analysis. This result was compatible with previous results indicating that no significant differences were observed in the adenylate and guanylate energy charges between the PD and the control iPSC‐derived cardiomyocytes.

## Discussion

Oxidative stress is associated with a variety of diseases, including cancer, neurodegeneration, and cardiovascular disease 11. In particular, diabetic cardiomyopathy and Duchenne muscular dystrophy have been associated with oxidative stress 12, 13. In addition, oxidative stress‐induced impairments of autophagy have been reported in a Duchenne muscular dystrophy mouse model (DMD^mdx^) 14. In line with this, disease‐specific iPSCs from Danon disease patients were recently used to show that oxidative stress is closely associated with the pathology of the disease 15.

There are a number of antioxidative defense mechanisms, such as NADPH/NADH and GSH/GSSG. NRF‐2 is a master regulator of antioxidative defense mechanisms. NRF‐2/ARE signaling has been shown to be associated with a variety of neurodegenerative diseases 16. Furthermore, activators of NRF‐2 have been investigated as therapeutic candidates in neurodegenerative disorders, such as Parkinson's disease 17.

Oxidative stress is also common to neurodegenerative and nonneurodegenerative lysosomal storage diseases 18. Our results show that oxidative stress may be associated with the cardiac complications of PD, both in vitro and in vivo. Moreover, we show that the NRF‐2/ARE‐mediated antioxidative stress mechanism is impaired in the cardiomyocytes and skeletal muscle cells of PD model mice.

Mitochondrial dysfunction has been suggested to be a disease‐modifying factor of PD. Fukuda et al. showed that mitochondrial dysfunction is present in skeletal muscle pathology and that oxidative stress may also be associated with PD pathogenesis 19. The etiology of the cardiac complications associated with PD remains unknown because it is difficult to obtain bioresources from this tissue compared with skeletal muscle, which is usually taken by muscle biopsy upon the diagnosis of PD. Our results are similar to observations made regarding skeletal muscle pathology and suggest that oxidative stress represents a major mechanistic driver of PD cardiomyopathy.

Lim et al. recently showed that mitochondrial oxidative stress induces the skeletal muscle pathology of PD and that Ca^2+^ homeostasis is dysregulated in the skeletal muscle of PD mice 20. The link between mitochondrial stress and oxidative stress is more clearly demonstrated by the results presented in this study. Using LC‐MS and CE‐MS analysis, we have shown that the metabolomic profile of PD iPSC‐derived cardiomyocytes differs from that of WT iPSC‐derived cardiomyocytes, which indicates that oxidative stress and mitochondrial dysfunction may be associated with the pathogenesis of cardiac complications in PD. In addition, we observed that the NRF‐2/ARE‐driven antioxidant mechanisms are downregulated in PD and may be associated with the underlying mechanisms of the disease.

It is important to note that our study was limited by the quality of the cardiomyocytes derived from iPSCs. We have used an efficient differentiation protocol, and the morphologies of both types of cardiomyocytes were similar after differentiation. However, despite the efficient differentiation from patient‐derived iPSCs, it is impossible to reproduce cardiac pathophysiology in vitro. In addition, cardiac complications are not prominent in PD mouse models and therefore make it difficult to study the cardiac complications associated with PD in vivo. In fact, oxidative stress is more prominent in skeletal muscle cells than in cardiomyocytes. However, we observed that NRF2 signaling was downregulated despite evidence of oxidative stress in the cardiomyocytes and skeletal muscle cells of the PD mouse model. Together, these results suggest that antioxidant mechanisms are likely downregulated in PD mice and highlight oxidative stress as a potential cause of cellular dysfunction.

Metabolomic profiling is a powerful tool to investigate global changes in cellular metabolism and is useful for investigating disease‐specific metabolic dysregulation, such as oxidative stress and mitochondrial impairment. By performing metabolomic profiling of iPSC‐derived cardiomyocytes, we have identified evidence of oxidative stress in a PD mouse model in vivo. Together, our results suggest that oxidative stress may be associated with cellular dysregulation in PD and could be a potential therapeutic target.

## Conclusion

Metabolomic analysis of PD iPSC‐derived cardiomyocytes revealed that oxidative stress and mitochondrial stress may underlie the cardiac pathology associated with PD. As seen in the metabolomic profiling, oxidative stress was also detected in skeletal muscle cells and cardiomyocytes in PD mice in vivo. Dysregulation of antioxidative stress mechanisms, such as NRF‐2, was also found in the skeletal muscle cells and cardiomyocytes of PD mice. Furthermore, we showed that the addition of an NRF‐2 activator could alleviate GSSG production and reduce ROS levels in cardiomyocytes derived from PD iPSCs. Together, these results suggest that oxidative stress could be potential therapeutic targets in PD.

## Author Contributions

Y. Sato: conception and design, manuscript writing; H.K.: financial support; T.H. and Y. Shimada: data analysis and interpretation; H.I. and T.O.: final approval of the manuscript.

## Disclosure of Potential Conflicts of Interest

T.O. and H.I. have active research support from Genzyme Japan Co., Ltd., and Shire Japan Co., Ltd. The other authors indicated no potential conflicts of interest.

## Supporting information

Supporting Information

Supporting Information

Supporting Information

Supporting Information
