# Supplementary material for: Metabolomic Profiling of Pompe Disease‐Induced Pluripotent Stem Cell‐Derived Cardiomyocytes Reveals That Oxidative Stress Is Associated with Cardiac and Skeletal Muscle Pathology
Source: Stem Cells Transl Med. 2016 Aug 18;6(1):31–9. doi: 10.5966/sctm.2015-0409 (PMC5442755; doi:10.5966/sctm.2015-0409)
Supplement: Supplementary file 1 — Supporting Information [file SCT3-6-031-s001.pdf]

Supplemental Figure – Ohashi et al.

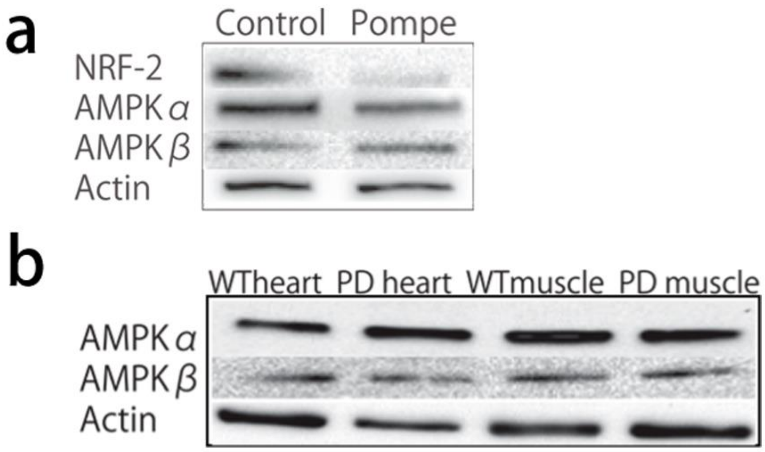

Figure S1. Western blot analysis. (a) PD iPSC-derived caridomyocytes. (b) Cardiomyocytes and skeletal muscle cells from PD mice.
